# Nursing Staff Salaries: Increase from the Top

**Published:** 1919-04-12

**Authors:** Alfred Whitham

**Affiliations:** Chairman. Becket Hospital, Barnsley.


*To the Editor of* The Hospital.

### Nursing Staff Salaries: Increase from the Top.

*To the Editor of* The Hospital.

Sir,—Those who peruse your weekly notes "Round the Hospitals," especially members of management committees, will note a growing desire throughout the country to do justice to the nursing profession. This desire is emphasised week by week from your reports of the increase of salaries at many hospitals, as well as reductions in the hours of service. One cannot but admit that these reforms are much overdue. Now that they are well before the public let us try to be fair to every section of the nursing profession. In the first place, may I point out that any hospital which is so fortunate as to have a real live matron at the head of it has a jewel that it ought to prize most highly? A payment in money itself does not represent the truth worth of such an one. A matron of this description is a real mother to those who are being trained under her care. She sees to it that each one under her charge is so trained and instructed in a nurse's duties that each pupil is a credit to her institution. The sisters and staff nurses under such a matron become imbued with her spirit of efficiency, hence probationers under these conditions have an opportunity of learning the profession in its higher sense. Therefore the time has come, if this spirit of high excellence is to be maintained, when much better salaries will have to be paid for it. Not from the bottom to the top, but rather from the top of the profession to the sisters and to the staff nurses. The probationers stand on a different plane from the general staff. They are really working students, and as such ought to be willing to make a considerable sacrifice during their training years, similar to what students in all other professions are called upon to make. May I suggest that this is a question that might well be considered by the British Hospitals Association? They ought to give a lead in this matter by advocating some uniform system of probationer payment. It is scarcely fair that poorly endowed hospitals should be compelled to compete with richly-endowed ones in procuring probationers. However, we must progress with the times, see that probationers are well cared for and efficiently trained, and then when certificated give them such salaries as will be commensurate to uphold the high calling they are engaged in and the high responsibility placed upon them.—Yours faithfully, Alfred Whitham, *Chairman*.

Becket Hospital, Barnsley.
April 5, 1919.

